# Does training on the WHO package of essential noncommunicable (PEN) disease interventions enhance consultation quality? A real-world assessment of adherence to PEN protocol in primary health centres in the Republic of Moldova

**DOI:** 10.1080/16549716.2023.2285619

**Published:** 2023-11-30

**Authors:** Jari Kempers, Cristina Rotaru, Alexandra Topa, Natalia Zarbailov, Ala Curteanu, Helen Prytherch

**Affiliations:** a European Health Economics Oy, Jyväskylä, Finland; b Independent Consultant, Chisinau, Republic of Moldova; c Public Medical Sanitary Institution, Territorial Medical Association of Botanica, Health Centre of Muncesti, Chisinau, Republic of Moldova; d Family Medicine Department, State University of Medicine and Pharmacy “Nicolae Testemitanu”, Chisinau, Republic of Moldova; e Healthy Life Project: Reducing the Burden of Noncommunicable Diseases, Chisinau, Republic of Moldova; f Research and Innovation Department, Mother and Child Health Institute, Chisinau, Republic of Moldova; g Swiss Centre for International Health, Swiss Tropical and Public Health Institute, Allschwil, Switzerland; h University of Basel, Basel, Switzerland

**Keywords:** Noncommunicable diseases, primary health care, WHO PEN protocol, PEN training, clinical protocol adherence

## Abstract

**Background:**

Noncommunicable diseases (NCDs) pose a significant global health challenge. Primary health centres are pivotal in addressing this challenge by providing essential care to NCD patients. The WHO Package of Essential Noncommunicable (PEN) disease interventions has been designed to enhance the quality of NCD consultations and ensure adherence to the protocol. This study investigates the effects of PEN training in Moldova.

**Objectives:**

The primary objective of this study is to assess the effects of training on WHO PEN on the quality of NCD consultations and adherence to the PEN protocol in a real -world setting in primary health centres in Moldova.

**Methods:**

An observational, cross-sectional study was conducted, comparing primary health centres where health personnel received PEN training, provided by the Healthy Life project, to those where such training was not provided. In total, 24 family doctors and 24 medical assistants were observed for 233 workdays and covering 2,166 NCD consultations.

**Results:**

Intervention primary health centres (PHCs) showed longer NCD consultation durations, with family doctors and medical assistants spending an added 1 minute 43 seconds and 3 minutes 10 seconds, respectively. These PHCs also reported a higher proportion of primary NCD consultations, indicating better screening for new NCD patients. Medical assistants in the intervention group took on a more pronounced role in NCD care. However, the findings also highlight the necessity to refine aspects of the PEN training, especially concerning follow-up consultations, risk assessments, and task delegation.

**Conclusions:**

The findings suggest that the PEN training contributed to improvement of both the quality of NCD consultations and adherence to the PEN protocol. Yet, there is a need for enhancing the identified aspects of the PEN training. The findings highlight the potential of PEN training in primary healthcare settings for improved NCD management.

## Introduction

Noncommunicable diseases (NCDs) account for a staggering 74% of all global deaths, translating to approximately 41 million lives lost annually [1]. 77% of NCD-related deaths occur in low- and middle-income countries, with 86% being premature [1]. Many of these countries, like the Republic of Moldova, struggle to achieve the Sustainable Development Goal target 3.4, which aims to reduce premature NCD mortality by a third between 2015 and 2030 [2]. In Moldova, NCDs account for nearly 90% of all deaths, with cardiovascular diseases causing 55% [3,4]. Men and rural population are disproportionately affected by NCDs, making them key groups for public health interventions [5]. Recent WHO studies indicated that in Moldova, 27.6% of adults aged 18–69 are current tobacco users and 63.2% consume alcohol, underscoring the persistently high prevalence of these risk factors for NCDs [5]. It is estimated that strengthening primary healthcare could prevent 60% of hypertension-related and 40% of diabetes-related hospitalisations through improved risk identification and management in Moldova [3].

The WHO Package of Essential Noncommunicable (PEN) disease interventions is a crucial step in enhancing primary healthcare’s approach to addressing NCDs. PEN prioritises a proactive approach to early detection, treatment, and management of NCDs. It emphasises simplified clinical protocols tailored for primary healthcare settings, enabling health personnel to offer timely, effective, comprehensive care and promote behaviour change for individuals at risk or already affected by NCDs [6,7]. Integral to the PEN’s approach is the establishment of a core set of indicators, facilitating ongoing monitoring of care quality and ultimate patient outcomes [8].

In 2015, recognising the challenge with NCDs, the Moldovan government embarked on a strategic initiative to integrate the WHO PEN protocol into its primary healthcare system [9]. This was accompanied by a push towards more integrated patient-centred care, essential for efficient use of limited healthcare resources of the country [10]. The national PEN protocol was officially introduced in 2018 [11]. Initially, a limited number of national PEN experts were trained [12]. The Healthy Life Project, supported by the Swiss Agency for Development and Cooperation, initiated a ‘training-of-trainers’ approach to accelerate the PEN training for primary healthcare professionals. In 2019, the project then launched a series of PEN trainings for family doctors and medical assistants to further scale up the implementation of the PEN protocol. To date, 1746 healthcare professionals have participated in these PEN trainings.

This study seeks to answer the following research question: Do primary health centres, where health personnel received training on the WHO PEN protocol from the Healthy Life Project, exhibit markers of improved NCD consultation quality and enhanced adherence to the protocol, compared to centres without such training? These findings can inform further refinements to the PEN training.

## Methods

### Study design

This is an observational, cross-sectional study comparing adherence to the WHO PEN protocol and assessing the quality of NCD consultations in rural primary health centres (PHCs) in Moldova. The study compares PHCs with family doctors (FDs) and medical assistants (MAs) who received PEN 5 Modules practical training through the Healthy Life Project to a control group of PHCs with FDs and MAs that were not trained on the PEN protocols by the Healthy Life Project. The focus of this study is on PEN consultations related to NCDs, with the primary objective being to assess markers quality and adherence to the PEN protocols rather than patient outcomes. The observations were conducted by trained observers who monitored NCD consultations performed by health personnel. Data collection was conducted from October to November 2022, capturing a snapshot of NCD consultations and PEN protocol adherence levels in both intervention and control facilities.

### Selection of primary health centres

Observations were conducted in four PHCs supported and trained by the Healthy Life Project (intervention group) and five similar PHCs not under the project’s support or training (control group). The intervention PHCs were selected based on their engagement with the Healthy Life Project, their rural location, and additionally, their demonstrated good performance in a hypertension services evaluation in 2018 [13]. The control PHCs were selected based on the following criteria: i) not supported by the Healthy Life Project, ii) rural location, iii) similar catchment populations, and iv) comparable numbers of health personnel (FDs and MAs). Table 1 shows details of the PHCs within the intervention and control groups and their pairing.

**Table 1.** Details of the primary health centres within the intervention and control groups, including their pairing, associated catchment populations, and the number of observed family doctors and medical assistants, as well as their observed consultations for all reasons and NCDs consultations.

Pair	Primary health centres and their groups	Catchment population	Family doctors	Family doctor consultations	NCD consultations	Medial assistants	Medical assistant consultations	NCD consultations
	Intervention group							
1	Briceni^†^	9,598	5	540	222	5	411	195
2	Criuleni	7,919	4	472	233	4	445	249
3	Marandeni, Falesti	2,672	1	116	40	1	93	46
4	Ciolacul Nou, Fălești	1,051	1	56	25	1	66	15
	**Total**	**21,240**	**11**	**1184**	**520**	**11**	**1015**	**505**
	Control group							
1	Riscani ^†^	11,142	2	212	88	2	196	95
1	Donduseni^†^	8,514	3	409	177	3	271	118
2	Nisporeni	11,903	5	529	212	5	430	171
3	Corlăteni, Rîșcani	5,476	2	236	97	2	248	100
4	Recea, Rîșcani	2,551	1	107	46	1	79	37
	**Total**	**39,586**	**13**	**1493**	**620**	**13**	**1224**	**521**

^†^Note that the Briceni PHC was paired with two primary health centres (PHCs), Donduseni and Riscani, due to challenges with the availability of family doctors in the Donduseni PHC during the study period. Therefore, a similar Riscani PHC was added as a control PHC, increasing the total number of control PHCs to five.

### Health personnel

The participation of health personnel in the study was voluntary. To increase their willingness to participate, a letter was sent from the MoH to the managers of PHCs, informing them about the importance of the study and requesting their cooperation. The WHO PEN protocol is designed for implementation by both FDs and MAs. Therefore, NCD consultations of both FDs and MAs were monitored onsite in all PHCs. In total, the research involved 24 FDs and 24 MAs, who were observed during 2,166 NCD consultations. The number of FDs, MAs and observed consultations for all reasons and NCDs consultations at both the intervention and control PHCs are shown in Table 1. The privacy of health personnel was maintained by excluding their names from the reports and by abstaining from conducting individual employee-level analyses.

### PEN training of the healthy life project

**Figure 1.**
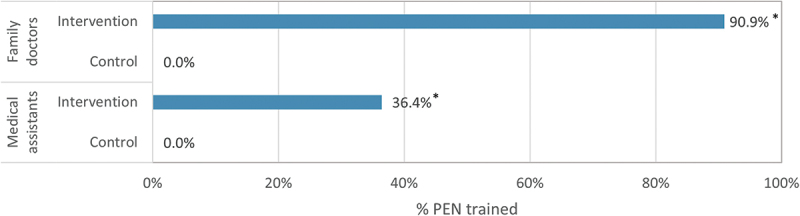
PEN training status of family doctors and medical assistants in intervention and control primary health centres. In the intervention primary health centres PEN training was provided by the Healthy Life Project.

In the intervention PHCs, nearly all FDs (90.9%) and 36.4% of MAs had received PEN training from the Healthy Life Project since 2018 (Figure 1). In contrast, in the control PHCs, neither FDs nor MAs participated in the PEN training provided by the Healthy Life Project. It is possible that health personnel from both the control and intervention PHCs have participated in PEN training approved by the MoH. However, this training is likely to have been less comprehensive, and participation from both groups was likely equal.

Statistically significant findings are indicated by * for p-values less than 0.05.

### Patient consent

The primary focus of this study was on NCD consultations, meaning that it was not a patient-centred study. Before consultations, all patients were verbally informed about the purpose of the research and assured that no personal information would be collected. Only patients’ age and gender were recorded, ensuring that the observation data could not be traced back to individual patients. Their verbal consent for observation in the consultation room was then obtained. If a patient declined, no observations were conducted. The Ethical Committee of the Ministry of Health granted ethical clearance for this study.

### Data collection forms

The study utilised purpose-built electronic forms that were developed and tested for this study: 1) a form for general information about health personnel, 2) a family doctor observation form, and 3) a medical assistant observation form. The observation forms underwent a two-stage validation: firstly, over a two-week period, three FDs and three MAs were observed during real consultations at two primary health centres in Chisinau, and secondly, academic collaborators from the State University of Medicine and Pharmacy ‘Nicolae Testemiţanu’ tested the forms in four simulated consultations. Prior to the commencement of the observations, the general information form was deployed to collect details such as age, role and the primary health centre in which the participants worked. Further, their medical education and any PEN training received through the Healthy Life project were documented. Lastly, data on their typical working hours and contract type were collected. This information was subsequently leveraged to enrich the analyses.

The observation forms, for both FDs and MAs, follow a similar structure. Each form begins by recording the start time and the current task that the FD or MA is engaged in. Depending on the type of activity, specific follow-up questions are presented. These questions are then filled out, and finally, the end time of the activity is recorded. The forms diverge when ‘Direct patient care’ is selected due to the different PEN protocol activities for FDs and MAs. In instances where a patient is diagnosed with an NCD or is suspected to have one, namely hypertension, ischaemic heart disease, diabetes or obesity, a checklist based on the WHO PEN protocol is prompted for completion. Moreover, reasons for the patients’ visit and potential further referrals are noted. All the forms were filled in only by the observers, using tablets. The forms were in the Romanian language. Health personnel or patients were not allowed to use the forms.

### Study size

The study size was determined as follows. To obtain a realistic representation of NCD consultations, the study aimed to observe at least 50% of the FDs and 30% of the MAs working in each PHCs. However, the actual number of health personnel observed was contingent upon the availability of FDs and MAs during the days when observers were present at the PHCs. No specific targets were set for the number of NCD consultations, as the volume and duration of these consultations were unknown beforehand.

### Statistical methods

The statistical methods implemented in this study encompassed the Chi-square statistic, Fisher’s Exact test, and ANOVA. When methodologically necessary, nonparametric equivalents were employed. A p-value threshold of less than 0.05 served as the criteria for statistical significance, with such values denoted by an asterisk (*) in the tables. Data analysis was carried out using IBM SPSS Statistics for Windows, Version 23.0. In the primary data analysis, various variables were considered for secondary cross-sections, with the goal of identifying clusters of NCD patients who received PEN protocol actions with varying frequencies. Both statistically significant and non-significant results, that contributed to the overall understanding of the findings and supported the conclusions, were included in this article. To prevent missing data, all questions in the PEN section of the forms were made mandatory.

## Results

### Characteristics of the NCD patients

The NCD patient groups of the intervention and control PHCs were comparable in terms of gender distribution, age distribution, and the number of health conditions consulted (Table 2). The differences between the two groups were minor, which suggests that the patient groups are similar enough to allow for a valid comparison when assessing the impact of the PEN training.

**Table 2.** Comparison of NCD patient characteristics in intervention and control primary health centres.

	Intervention		Control		
Characteristics	n	%	n	%	Δ%
**Sex**					
Male	392	38.2	441	38.7	−0.4
Female	633	61.8	700	61.3	0.4
**Age of patient**					
18–45 years old	50	4.9	43	3.8	1.1
45–70 years old	728	71.0	803	70.4	0.6
over 70 years old	247	24.1	295	25.9	−1.8
**Number of health conditions consulted**					
Single health condition	491	47.9*	596	52.2*	−4.3*
Multiple health conditions	534	52.1*	545	47.8*	4.3*
**Health conditions consulted**					
**NCD**					
Hypertensive diseases	950	92.7	1048	91.8	0.8
Ischemic heart diseases	385	37.6	393	34.4	3.1
Diabetes	293	28.6	326	28.6	0.0
Overweight, obesity	203	19.8*	92	8.1*	11.7*
**Non-NCD comorbidities**					
Musculoskeletal system, diseases of	147	14.3	197	17.3	−2.9
Digestive system, diseases of	105	10.2	143	12.5	−2.3
Visual system, diseases of	102	10.0*	79	6.9*	3.0*
Respiratory system, diseases of	80	7.8	64	5.6	2.2
Endocrine diseases (excluding diabetes)	77	7.5*	50	4.4*	3.1*
Sexual and reproductive health problems	5	0.5	3	0.3	0.2
Other	179	17.5	179	15.7	1.7
**Total NCD consultations**	**1025**	**100.0**	**1141**	**100.0**	

Statistically significant findings are indicated by * for p-values less than 0.05.

### Duration of NCD consultations

**Figure 2.**
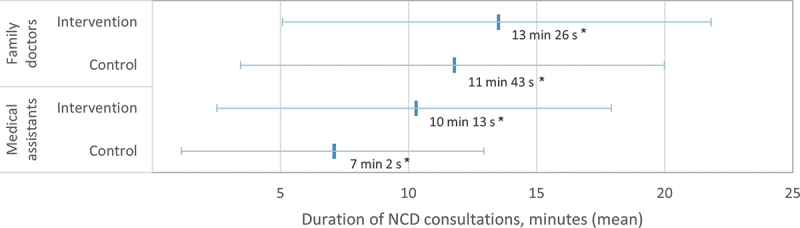
The duration of the NCD consultations per patient by family doctors and medical assistants in intervention and control primary health centres.

NCD consultations in the intervention PHCs were significantly longer for both FDs and MAs in comparison to their counterparts in the control PHCs (Figure 2). Specifically, FDs in the intervention PHCs spent an additional 1 minute and 43 seconds per NCD consultation, while MAs expended an extra 3 minutes and 10 seconds, compared to their peers in the control PHCs. The longer time spent per NCD patient suggests better adherence to the PEN protocol in the intervention PHCs, potentially leading to better quality NCD consultations compared to the control PHCs.

Statistically significant findings are indicated by * for p-values less than 0.05.

### Primary and follow-up NCD consultations

**Figure 3.**
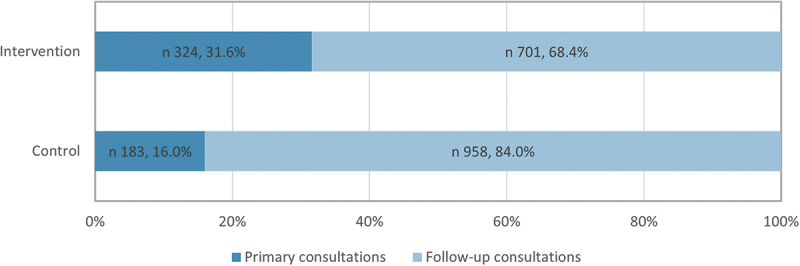
Distribution of primary and follow-up visits in observed NCD consultations in intervention and control primary health centres.

A significantly higher percentage of NCD consultations in the intervention PHCs were primary consultations (Figure 3). This indicates that the intervention PHCs were more effective in identifying new NCD patients in comparison with their control counterparts. The term ‘primary NCD consultations’ in this study encompasses visits during which an NCD was identified and treated for the first time. Conversely, ‘follow-up NCD consultations’ signify recurring visits during which the treatment of a previously diagnosed NCD was continued.

### Medical history taking and physical examination by family doctors

During the primary NCD consultations, FDs from intervention PHCs demonstrated better performance in medical history taking (PEN action 1) and notably better in the physical examination of the cardiovascular system (PEN action 2) than their counterparts in the control group (Table 3). However, there is room for improvement in follow-up NCD consultations. Interestingly, during the follow-up consultations, the control PHCs outperformed the intervention PHCs in both medical history taking and physical examination.

**Table 3.** Medical history taking and physical examination (PEN actions 1 and 2) performed by family doctors during primary and follow-up NCD consultations in intervention and control primary health centres.

	Intervention		Control		
	n	%	n	%	Δ%
**Primary NCD consultations**					
**Action 1: Medical history taking**					
Diagnosed chronic diseases	129	94.2	95	88.8	5.4
NCD symptoms	130	94.9	103	96.3	−1.4
Medicines taken	120	87.6	90	84.1	3.5
**Action 2: Physical examination**					
Cardiovascular system (apex beat, heart auscultation, peripheral oedema)	93	67.9*	52	48.6*	19.3*
Evaluation/referral to investigations and laboratory tests	90	65.7	80	74.8	−9.1
**Total primary NCD consultations by FDs**	**137**	**100.0**	**107**	**100.0**	
**Follow-up NCD consultations**					
**Action 1: Medical history taking**					
Diagnosed chronic diseases	247	64.5*	421	82.1*	−17.6*
NCD symptoms	273	71.3*	448	87.3*	−16.1*
Medicines taken	321	83.8	422	82.3	1.6
**Action 2: Physical examination**					
Cardiovascular system (apex beat, heart auscultation, peripheral oedema)	181	47.3*	303	59.1*	−11.8*
Evaluation/referral to investigations and laboratory tests	240	62.7	336	65.5	−2.8
**Total follow-up NCD consultations by FDs**	**383**	**100.0**	**513**	**100.0**	

Statistically significant findings are indicated by * for p-values less than 0.05.

### Risk factor assessments by family doctors and medical assistants

In terms of risk factor assessment during primary and follow-up NCD consultations, clear differences emerged between the intervention and control PHCs with respect to adherence to the PEN protocol (Table 4). In primary NCD consultations, FDs in the intervention group were more frequently assessing diet and physical activity compared to FDs in the control group. However, with alcohol and tobacco use assessments, FDs in the control group were conducting these more frequently. During follow-up NCD consultations, FDs in the intervention group were less likely to conduct assessments in all categories; diet, physical activity, alcohol and tobacco use, compared to FDs in the control PHCs.

**Table 4.** Risk factor assessment (PEN action 1) carried out by family doctors and medical assistants during primary and follow-up NCD consultations in the intervention and control primary health centres.

	Intervention		Control		
	n	%	n	%	Δ%
**Family Doctors**					
**Primary NCD consultations**					
Diet	105	76.6	77	72.0	4.6
Physical activity	92	67.2	59	55.1	12.1
Alcohol use	3	2.2	10	9.3	−7.1
Tobacco use	2	1.5	9	8.4	−6.9
**Total**	**137**	**100.0**	**107**	**100.0**	
**Follow-up NCD consultations**					
Diet	246	64.2	376	73.3	−9.1
Physical activity	193	50.4	309	60.2	−9.8
Alcohol use	45	11.7	128	25.0	−13.3
Tobacco use	48	12.5	118	23.0	−10.5
**Total**	**383**	**100.0**	**513**	**100.0**	
**Medical Assistants**					
**Primary NCD consultations**					
Diet	144	77.0	23	30.3	46.7
Physical activity	149	79.7	41	53.9	25.8
Alcohol use	18	9.6	5	6.6	3.0
Tobacco use	19	10.2	4	5.3	4.9
**Total**	**187**	**100.0**	**76**	**100.0**	
**Follow-up NCD consultations**					
Diet	186	58.5	116	26.1	32.4
Physical activity	175	55.0	95	21.3	33.7
Alcohol use	71	22.3	17	3.8	18.5
Tobacco use	65	20.4	12	2.7	17.7
**Total**	**318**	**100.0**	**445**	**100.0**	

In both primary and follow-up NCD consultations, MAs from the intervention group showed greater adherence to the PEN protocol than those in the control group. Specifically, during primary consultations, a higher proportion of intervention MAs assessed diet and physical activity, as well as alcohol and tobacco use. Also in follow-up consultations, the intervention MAs maintained a notable lead, particularly with regard to alcohol and tobacco use assessments. This points to a consistent pattern of more thorough risk factor assessment by the intervention group MAs, as compared to their counterparts in the control PHCs.

### Physical examinations by family doctors and medical assistants

The results related to physical examinations were mixed, revealing different levels of adherence to PEN action 2 across the PHC groups. During primary NCD consultations, FDs in the intervention PHCs conducted anthropometric measures and blood pressure and heart rate measurement less frequently than those in the control PHCs (Table 5). FDs in the intervention PHCs displayed higher adherence in conducting cardiovascular system examinations compared to those in the control PHCs during primary NCD consultations; however, this trend was reversed in the follow-up consultations. MAs from the intervention PHCs consistently outperformed their counterparts in conducting anthropometric measures.

**Table 5.** Physical examinations; anthropometric measures, blood pressure and heart rate and cardiovascular system examinations (PEN action 2) performed by family doctors and medical assistants during primary and follow-up NCD consultations in the intervention and control primary health centres.

	Intervention				Control				Δ%	
	FD		MA		FD		MA		FD	MA
	n	%	n	%	n	%	n	%	%	%
**Primary NCD consultations**										
Anthropometric measures	17	12.4	36	19.3	24	22.4*	8	10.5*	-10.0	8.8
Blood pressure and heart rate	89	65.0*	170	90.9*	102	95.3	68	89.5	-30.3	1.4
Cardiovascular system (apex beat, heart auscultation, peripheral oedema), FDs only	93	67.9*			52	48.6*			19.3*	
**Total primary NCD consultations**	**137**	**100.0**	**187**	**100.0**	**107**	**100.0**	**76**	**100.0**		
**Follow-up NCD consultations**										
Anthropometric measures	47	12.3*	104	32.7*	49	9.6*	15	3.4*	2.7	29.3
Blood pressure and heart rate	282	73.6	240	75.5	467	91.0*	340	76.4*	-17.4	-0.9
Cardiovascular system (apex beat, heart auscultation, peripheral oedema), FDs only	181	47.3*			303	59.1*			-11.8*	
**Total follow-up NCD consultations**	**383**	**100.0**	**318**	**100.0**	**513**	**100.0**	**445**	**100.0**		

Statistically significant findings are indicated by * for p-values less than 0.05.

### Assessment of cardiovascular and diabetes risks by family doctors and medical assistants

The WHO PEN protocol recommends periodic risk assessment for cardiovascular disease and diabetes in NCD patients, using the cardiovascular risk (SCORE) and the Finnish Diabetes Risk Score (FINDRISC) tools [xi]. According to the National PEN protocol of Moldova, SCORE assessments should be done during every NCD consultation, except for patients who already developed cerebrovascular disease, chronic kidney disease, or diabetes [xi]. The FINDRISC is recommended every five years for patients with low to moderate diabetes risks and every three years for high-risk patients during follow-up NCD consultations.

The results from the risk assessments present a mixed picture. During primary NCD consultations, both FDs and MAs in the intervention PHCs were less consistent in their use of the SCORE and FINDRISC risk assessments than their counterparts in the control PHCs (Table 6). However, during follow-up NCD consultations, FDs in the intervention PHCs demonstrated better adherence to the SCORE assessments compared to the control PHCs. The frequency of FINDRISC assessments conducted by MAs is notably low, with a negligible percentage difference.

**Table 6.** Cardiovascular and diabetes risk assessment (PEN action 3) performed by family doctors and medical assistants during primary and follow-up NCD consultations in the intervention and control primary health centres.

	Intervention				Control				Δ%	
	FD		MA		FD		MA		FD	MA
	n	%	n	%	n	%	n	%	%	%
**Primary NCD consultations**										
Cardiovascular risk (SCORE)	15	16.1	17	11.8	21	32.8	10	27.8	−16.7	−16.0
Diabetes risk (FINDRISC)	7	6.9	5	3.6	12	15.0*	2	3.5*	−8.1	0.1
**Follow-up consultations**										
Cardiovascular risk (SCORE)	50	21.6*	12	7.0*	45	13.6*	11	3.5*	8.0	2.0
Diabetes risk (FINDRISC)	9	3.5*	1	0.4*	12	3.5	5	1.5	0.0	−1.1

Statistically significant findings are indicated by * for p-values less than 0.05.

Importantly, the findings indicate that both risk assessments, SCORE and FINDRISC, were significantly underutilised in both intervention and control PHCs. It was not feasible to determine what the optimal number of risk assessments that should have been conducted for the sample, as this varies based on patient risk profiles, symptom presence, complications, and the clinical judgement of the health personnel. Nevertheless, based on a conservative estimate, 80% of NCD patients do not have cerebrovascular disease, chronic kidney disease, or diabetes. These patients typically have two NCD consultations annually. Therefore, one would expect at least 400 SCORE assessments in the observed NCD consultations. However, only about 90 SCORE assessments were conducted across both intervention and control PHCs. For FINDRISC, considering 40% of NCD patients are at diabetes risk and have biannual consultations, approximately 5% of the observed NCD consultations should include a FINDRISC assessment. This equals to 50 evaluations across both PHC groups, but only 27 were conducted.

### Scheduling of NCD-related follow-up consultations by family doctors

FDs in intervention PHCs were less likely to schedule follow-up NCD consultations than those in control HCs (Table 7). Intervention PHCs largely adhered to the PEN protocol, scheduling follow-ups within 3 months. On the contrary, FDs in the control PHCs scheduled a significant portion the next consultations within a 3–6-month timeframe, and therefore deviating from the PEN protocol.

**Table 7.** Scheduling of NCD-related follow-up consultations for NCD patients by family doctors in the intervention and control primary health centres.

	Intervention		Control		
	n	%	n	%	Δ%
**Did the FD schedule a follow up consultation (NCD related) at the primary health centre?**					
Yes	369	71.0*	491	79.2*	−8.2*
No	151	29.0*	129	20.8*	8.2*
**Total NCD consultations by FDs**	**520**	**100.0**	**620**	**100.0**	
**When was the follow-up consultation scheduled?**					
Less than 3 months	301	81.6*	265	54.0*	27.6*
3–6 months	67	18.2*	225	45.8*	−27.7*
More than 6 months	1	0.3	1	0.2	0.1
**Total NCD-related follow-up consultations**	**369**	**100.0**	**491**	**100.0**	

Statistically significant findings are indicated by * for p-values less than 0.05.

### Referral practices of family doctors

**Figure 4.**
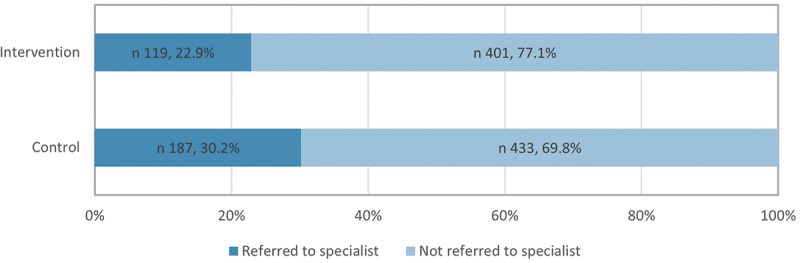
Referrals of NCD patients to a specialist or hospital by family doctors in the intervention and control primary health centres.

FDs from the intervention PHCs exhibited a more conservative approach in their referral practices, referring 7.3% fewer NCD patients to specialists or hospitals than FDs in the control PHCs (Figure 4). Most of these referrals directed patients to either the same institution or neighbouring ones and the remainder were sent to district and national level institutions. While cardiologists, endocrinologists, neurologists and ophthalmologists were the most common specialists to whom patients were referred, there was no significant difference in the choice of specialist between the intervention and control PHCs. In addition, FDs from the intervention PHCs were slightly less likely to refer NCD patients to further investigations or laboratory tests at the PHCs.

## Discussion

The results of this study present a mixed picture regarding the effects of training on the WHO PEN protocol provided by the Healthy Life Project. This discussion reflects a balanced assessment of all findings, highlighting both positive and negative results. The findings suggest that the PEN training contributed to improvements in several aspects of NCD care. In the intervention primary health centres (PHCs), where most family doctors (FDs) underwent PEN training, there were markers of improved quality in NCD consultations and enhanced adherence to the PEN protocol. Notably, the duration of NCD consultations was longer in the intervention PHCs; FDs spent an additional 1 minute and 43 seconds, and medical assistants (MAs) spent 3 minutes and 10 seconds longer with NCD patients, compared to their counterparts in the control PHCs. The intervention PHCs also had a higher proportion of primary NCD consultations, suggesting more effective identification of new NCD patients. In the intervention PHCs, MAs assumed a more prominent role in NCD patient care, particularly in the assessment of risk factors and physical examinations during both primary and follow-up consultations. FDs from the intervention PHCs referred 7.3% fewer NCD patients to specialists or hospitals compared to those in the control PHCs. On the other hand, some findings indicate that despite the PEN training, there are several areas where the intervention PHCs did not demonstrate improvements. Surprisingly, FDs in the intervention PHCs performed more poorly during follow-up NCD consultations in aspects such as medical history-taking, physical examinations, risk factor assessments, and evaluations of the cardiovascular system compared to their counterparts in the control PHCs. Moreover, both SCORE and FINDRISC risk assessments remained significantly underutilised in both groups. While MAs took on a more active role in risk assessments and physical examinations, as is anticipated by the PEN approach, the expected corresponding reduction in FDs’ tasks was not observed.

There are clear indicators that the PEN training provided improved some aspects of NCD services in primary healthcare settings. The extended consultation time by both FDs and MAs allows for a more thorough clinical evaluation, better adherence to the PEN protocol, and consequently, a potential improvement in the quality of NCD services. The higher proportion of primary NCD consultations in the intervention PHCs indicates more effective screening and identification of new NCD patients, which is essential for early detection, treatment, and management of NCDs. This outcome aligns with the expectation set by the matched clinic type, location, and catchment population of the intervention and control PHCs, underlining the efficacy of the PEN training in enhancing the proactive identification of NCD cases within routine clinical practice. MAs assumed a more prominent role in NCD patient care, which is welcomed by the human resource-constrained healthcare system of Moldova. Moreover, MAs adhered better to the PEN protocol and were more thorough in risk factor assessments, contributing to better risk management and prevention practices. Interestingly, this shift occurred even though all MAs had not undergone PEN training, indicating that PEN-trained FDs may have encouraged MAs to take on more responsibilities. In the intervention PHCs, FDs referred notably fewer NCD patients to specialists or hospitals. This gatekeeping function of FDs is central in the resource-constrained healthcare system of Moldova. The PEN training enhanced their confidence and competence in managing NCD cases and provided clearer guidance on the criteria for NCD referrals.

The findings also highlight the need for enhancement and refocusing of some aspects of the PEN training, especially in relation to follow-up consultations. Generally, PEN-trained FDs conduct primary NCD consultations well. However, this focus seems to lead them to develop overconfidence in their understanding of the patient’s clinical condition, subsequently neglecting necessary examinations during follow-up consultations. Therefore, PEN training should stress the importance of adhering to the protocol and conducting all required examinations during follow-up consultations as well. The training should also emphasise the systematic use of SCORE and FINDRISC assessments, clearly stating when and to which patients these assessments should be administered. The increased responsibilities of MAs did not result in a corresponding reduction of tasks for FDs. This potential task duplication between FDs and MAs could be due to inadequate communication or mistrust between cadres of healthcare workers. Therefore, PEN training should clearly outline the tasks that MAs are competent to perform. This would make FDs more comfortable delegating responsibilities and reduce the need to double-check tasks already completed by MAs.

In comparison to another study on WHO PEN capacity building for NCD management in Ukraine, our findings align with the observation that such capacity building enhances health personnel’s proficiency in detecting and assessing risks and risk factors associated with NCDs [14]. However, the Ukrainian study emphasised that improved treatment outcomes were not sustained over a two-year follow-up period, highlighting the necessity for continuous medical education on PEN. Another study from Gaza reported that health personnel trained on WHO PEN exhibited a heightened awareness of NCD management, referral criteria, and the utilisation of monitoring tools [15]. Additionally, research conducted in the Democratic People’s Republic of Korea concluded that the implementation of the WHO PEN protocol bolstered risk management in their polyclinics [16].

This study has several limitations that should be considered when interpreting its findings. First, the voluntary non-randomised participation of FDs and MAs introduces a selection bias, as those who chose to participate may be more inclined to adhere to the PEN protocol. Second, health personnel were aware that they were being observed and had been informed in advance about the timing of these observations. This could potentially lead to more diligent performance and an overestimation of real-world adherence to the PEN protocol. Third, not all FDs and MAs in the intervention PHCs had undergone PEN training, which might introduce biases that affect the validity of the study outcomes. Finally, the study’s cross-sectional design captures only a snapshot in time, making it difficult to track changes in NCD consultation practices and PEN protocol adherence, or to establish causal relationships between PEN training and healthcare outcomes.

## Conclusion

This study offers a detailed assessment of the impact of the WHO PEN protocol training, provided by the Healthy Life Project, on the quality of NCD consultations and adherence to the PEN protocol in primary health centres in Moldova. While the findings are somewhat mixed, the balance of evidence and a holistic view of the results suggest that the PEN training improved both the quality of NCD consultations and adherence to the PEN protocol. This improvement is particularly notable in terms of longer consultation durations, increased rates of primary NCD consultations, and more effective utilisation of medical assistants in NCD care. The study also indicates that family doctors in the intervention PHCs made fewer referrals to specialists or hospitals. Hence, reinforcing the pivotal gatekeeper role of FDs in a context like Moldova’s. On the other hand, the study also highlights the need for refinement in certain aspects of the PEN training, particularly concerning follow-up consultations, risk assessments, and effective task delegation. The study demonstrates that PEN training has the potential to significantly improve NCD management in primary healthcare settings.

## Paper context

The WHO’s PEN protocol aims to improve the prevention, early diagnosis, and management of non-communicable diseases (NCDs). This study investigates the effects of PEN training in primary health centres in Moldova. The findings suggest that the PEN training contributed to improvement of the quality of NCD consultations and adherence to the PEN protocol. However, there are areas in the training, particularly follow-up consultations and task delegation, that require refinement.
